# A novel panel of clinically relevant miRNAs signature accurately differentiates oral cancer from normal mucosa

**DOI:** 10.3389/fonc.2022.1072579

**Published:** 2022-12-01

**Authors:** Nikolay Mehterov, Andrea Sacconi, Claudio Pulito, Boyan Vladimirov, Georgi Haralanov, Dimitar Pazardjikliev, Boyan Nonchev, Ioana Berindan-Neagoe, Giovanni Blandino, Victoria Sarafian

**Affiliations:** ^1^ Department of Medical Biology, Medical University-Plovdiv, Plovdiv, Bulgaria; ^2^ Research Institute, Medical University-Plovdiv, Plovdiv, Bulgaria; ^3^ UOSD Clinical Trial Center, Biostatistics and Bioinformatics, IRCCS Regina Elena National Cancer Institute, Rome, Italy; ^4^ Translational Oncology Research Unit, Department of Research, Advanced Diagnostic, and Technological Innovation, IRCCS, Regina Elena National Cancer Institute, Rome, Italy; ^5^ Department of Maxillofacial Surgery, Medical University-Plovdiv, Plovdiv, Bulgaria; ^6^ Department of Otorhinolaryngology, Medical University-Plovdiv, Plovdiv, Bulgaria; ^7^ Department of Endocrinology, Medical University-Plovdiv, Plovdiv, Bulgaria; ^8^ Research Center for Functional Genomics, Biomedicine and Translational Medicine, “Iuliu Hatieganu” University of Medicine and Pharmacy, Cluj-Napoca, Romania

**Keywords:** oral squamous cell carcinoma, biomarker, miRNA, mRNA, overall survival, disease-free survival

## Abstract

**Introduction:**

Although a considerable body of knowledge has been accumulated regarding the early diagnosis and treatment of oral squamous cell carcinoma (OSCC), its survival rates have not improved over the last decades. Thus, deciphering the molecular mechanisms governing oral cancer will support the development of even better diagnostic and therapeutic strategies. Previous studies have linked aberrantly expressed microRNAs (miRNAs) with the development of OSCC.

**Methods:**

We combined bioinformatical and molecular methods to identify miRNAs with possible clinical significance as biomarkers in OSCC. A set of 10 miRNAs were selected via an in silico approach by analysing the 3’untranslated regions (3’UTRs) of cancer-related mRNAs such as FLRT2, NTRK3, and SLC8A1, TFCP2L1 and etc. RT-qPCR was used to compare the expression of in silico identified miRNAs in OSCC and normal tissues (n=32).

**Results:**

Among the screened miRNAs, miR-21-5p (p < 0.0001), miR-93-5p (p < 0.0197), miR-146b-5p (p <0.0012), miR-155-5p (p < 0.0001), miR-182-5p (p < 0.0001) were significantly overexpressed, whereas miR-133b (p < 0.05) was significantly downregulated in OSCC tissues, a scenario confirmed in two additional OSCC validation cohorts: Regina Elena National Cancer Institute (IRE cohort, N=74) and The Cancer Genome Atlas Data Portal (TCGA cohort, N=354). Initial stage tumors (T1, T2) expressed significantly higher levels of miR-133b (p < 0.0004) compared to more advanced ones (T3, T4). Also, we identified miR-93-5p (p < 0.0003), miR-133b (p < 0.0017) and miR-155-5p (p < 0.0004) as correlated with HPV-induced OSCC. The high expression of these 6 miRNAs as a signature predicted shorter disease-free survival (DFS) and could efficiently distinguish OSCC cases from healthy controls with areas under the curve (AUC) of 0.91 with sensitivity and specificity of 0.98 and 0.6, respectively. Further target identification analysis revealed enrichment of genes involved in FOXO, longevity, glycan biosynthesis and p53 cancer-related signaling pathways. Also, the selected targets were underexpressed in OSCC tissues and showed clinical significance related to overall survival (OS) and DFS.

**Discussion:**

Our results demonstrate that a novel panel consisting of miR-21-5p, miR-93-5p, miR-133b, miR-146b-5p, miR-155-5p and miR-182-5p could be used as OSCC-specific molecular signature with diagnostic and prognostic significance related to OS and DFS.

## Introduction

Oral squamous cell carcinoma (OSCC) is the most common malignant tumor of the oral cavity, accounting for more than 95% of these cancers (1). The main risk factors leading to the development of OSCC are connected with age, tobacco use, alcohol consumption, and human papillomavirus (HPV) infection (2–4). Despite the recent advances in the field related to a better quality of life and treatment, the 5-year OS has not changed in the last decade and remains around 50% (5). Late diagnosis, high rate of recurrence, local invasiveness, and high risk of lymph node metastasis are among the reasons for the unchanged mortality rate (6). Treatment modalities of oral cancer include surgery, radiotherapy and chemotherapy, or a combination of these (7), with surgery remaining the mainstay of treatment for curable, resectable disease (8). Unfortunately, there has been only limited advances in targeted therapy for oral cancer. The first step in that direction was the approval of the use of a monoclonal antibody against EGFR, cetuximab, in oral cancer treatment (9). It was followed much later by the introduction of anti-PD1 agents (pembrolizumab and nivolumab) in the treatment of HNSCC (10, 11). Still, a lot of efforts are directed towards the elucidation of the altered genetic landscape of oral cancer, especially with the use of techniques as NGS, and databases like TCGA. As a result, data about frequently mutated genes has been accumulated, especially TP53, FAT1, CDKN2A, NOTCH1, PIK3CA, CASP8, HRAS, among others (12). Genetic studies are expected to pave the way to more effective targeted therapies. It still remains a fact that if treatment is performed at the T1 or T2 stages the percentage of cases without recurrences increases up to 84.6% (13). Therefore, to reduce OSCC-related deaths it is necessary to identify new OSCC-specific biomarkers for early diagnosis as well.

In the last decade, a class of small protein non-coding RNA molecules called miRNA have gained particular attention as major regulators of gene expression at the posttranscriptional level. They function either by degradation of their target transcripts or by translational repression of the synthesized protein. Recent studies suggest that 30 to 80% of all human genes are predicted to act as targets of miRNAs, making the latter master regulators of fundamental cellular processes such as cell cycle, differentiation, and apoptosis (14). As a result of their pivotal role, the dysregulation of miRNAs is potentially dangerous and may switch to carcinogenesis in otherwise healthy tissues (15, 16). The global transcription profile of tumorous versus normal tissues shows drastic transcription changes, characterized by activation of oncogenic miRNAs and inhibition of tumor suppressor ones. Within oral cancer miRNA expression patterns are associated with cell proliferation and apoptosis (miR-99a, -26a, -451, -128), metastasis development (miR-141, -200c) and radio- and chemotherapy resistance (miR-125b, -494-3p, -222) (17–24). The proof that miRNAs play an essential role in the transition of oral leukoplakia into invasive OSCC is reported by Cervigne and colleagues. They identified a group of miRNA (miR-146b, -181b, -21, -345, -518b, -520g, -649 and -184) commonly expressed in both conditions and thus considered them as early detectable events in oral tumor progression (25). Therefore, the identification of novel miRNA biomarkers will shed new light on the molecular mechanisms that govern this type of malignancy.

In a previous study, we have shown that salivary miR-30c-5p can efficiently discriminate patients of two independent oral cancer cohorts from the healthy group (26). In addition, we identified 4 out of 35 deregulated miRNAs that can actually predict local recurrence independently from prognostic clinical variables, either when considered individually or as a signature (27).

In the present study, we used a bioinformatical approach that led to the selection of 10 miRNAs that have binding sites in 3’UTRs of oral and other cancer-related mRNAs. Their transcription levels were further assessed in normal and tumor oral tissues obtained from OSCC patients. Among them, miR-21-5p, miR-93-5p, miR-146b-5p, miR-155-5p, and miR-182-5p were upregulated, whereas miR-133b was downregulated in OSCC tissues, compared to their normal adjacent tissue The additional validation in two other patient cohorts confirmed the same pattern of expression. We showed that miR-133b is predominantly expressed in T1/T2 tumor stages compared to T3/T4, whereas miR-93-5p, miR-133b, and miR-155-5p could be used to distinguish HPV-induced tumors from non-HPV-induced. ROC curve analysis demonstrated the properties of these miRNAs as a biomarker signature since they were able to differentiate between OSCC cases and controls and also predict shorter DFS. The target identification analysis revealed enrichment of miRNA signature validated targets (n=55) related to FOXO, longevity, glycan biosynthesis, p53 cancer-associated signaling pathways, etc. The following statistical analysis (Fold Change<-1.5 and Pearson R<-0.3) resulted in a final list of 14 genes, negatively correlated to the miRNA signature and prognostic for both OS and DFS when considered as a group. We believe that the novel data presented here could allow the use of these miRNAs as biomarkers with prognostic clinical significance for OS and DFS in OSCC.

## Materials and methods

### Clinical samples and patient characteristics

Newly diagnosed OSCC patients (n=34) from University Hospital St. George Plovdiv (UHSG cohort) between 2013-2016 were included in the study. Tumor tissue and non-tumorous oral mucosa at least 2 cm away from the tumor site were collected from each patient after signed informed consent. Immediately after surgery, the specimens were immersed in PAXgene Tissue Containers (Qiagen) according to the manufacturer’s recommendation and stored at − 80°C before RNA extraction. The tissue samples were obtained from the following sites: tongue (7), mouth floor (14), buccal mucosa (2), gingiva (10), and retromolar triangle (1). The histology of the tissues was evaluated by two independent pathologists. None of the patients had undergone neither radiotherapy nor chemotherapy before surgical treatment. Clinical and demographic parameters including age, sex, social history, pathological features, TMN stage, therapy, and OS were prospectively collected (**Table 1**). The patients were classified according to the 2002 Union for International Cancer Control Tumor-Node-Metastasis (TNM) staging system. The study was approved by the Institutional Ethics Committee of Medical University-Plovdiv (Protocol №1/25.02.2016).

**Table 1:** Clinical and demographic characteristics of the UHSG patient cohort.

Patient code	Gender	Age (years)^a^	Tumour location	Histology	T^b^	N^c^	M^d^	Differentiation	HPV		Smoker	Alcohol	Therapy (Radio, Chemo, Surgical)	Overall survival^e^ (weeks/months)	Vital Status
									16	18					
OC1	M	61	Floor of mouth	SCC	4	1	x	Moderate	–	–	Yes	Yes	Chem+Rad	12m	Dead
OC3	M	64	Lower jaw	SCC	4	2	1	Well	–	–	Yes	Yes	Surg+Chem	5m	Dead
OC4	M	66	Floor of mouth	SCC	2	1	x	Moderate	–	–	Yes	Yes	No	7m	Dead
OC5	M	52	Lower jaw	SCC	4	2	x	Well	–	–	Yes	Yes	Surg	3m	Dead
OC6	M	69	Floor of mouth	SCC	1	0	x	Well	–	–	Yes	Yes	Surg	30m	Dead
OC7	M	71	Lower jaw	SCC	4	1	x	Moderate	–	–	Former	No	No	0m	Dead
OC8	M	60	Tongue	SCC	4	1	x	Moderate	–	–	Yes	Yes	Surg+Chem	7m2w	Dead
OC9	M	72	Floor of mouth	SCC	4	1	x	Moderate	–	–	Yes	Yes	Surg	20m+	Dead
OC10	M	47	Floor of mouth and tongue	SCC	2	0	x	Moderate	–	–	Yes	Yes	Surg+Chem+Rad	7m	Dead
OC11	F	48	Floor of mouth	SCC	4	1	x	Moderate	–	–	Yes	No	Surg+Rad	41m+	Alive
OC12	M	54	Tongue and bottom of the mouth	SCC	3	0	x	Poor	–	–	Yes	Yes	Rad	12m	Dead
OC13	M	52	Floor of mouth	SCC	2	1	x	Moderate	–	–	Yes	Yes	Surg	42m+	Alive
OC14	M	65	Floor of mouth	SCC	1	0	x	Poor	–	–	Yes	Yes	Surg	–	–
OC15	M	76	Gums of the frontal area of the mandible	SCC	2	1	x	Well	–	–	No	No	Rad	7m	Dead
OC16	M	69	Lower jaw	SCC	4	0	x	Well	–	–	Yes	Yes	Surg+Rad	41m+	Alive
OC17	M	47	Tongue	SCC	3	0	x	Moderate	–	–	Yes	Yes	Surg+Rad+Chem	28m	Dead
OC18	F	76	Tongue	SCC	2	0	x	Moderate	–	–	Yes	No	Surg	28+	Alive
OC19	M	59	Lower jaw	SCC	4	2	x	Moderate	–	–	Yes	Yes	Surg	2w	Dead
OC20	M	58	Lower jaw	SCC	4	2	x	Moderate	–	–	Yes	Yes	No	3m	Dead
OC21	M	60	Upper jaw	SCC	4	2	x	Moderate	–	–	Yes	Yes	Surg+Rad	7m	Dead
OC22	M	64	Tongue	SCC	2	0	x	Well	–	–	Yes	Yes	Surg	5m	Dead
OC23	M	56	Floor of mouth	SCC	3	1	x	Moderate	–	–	Yes	Yes	Rad	6m	Dead
OC24	M	60	Floor of mouth	SCC	2	0	x	Moderate	–	–	Yes	No	No	2m2w	Dead
OC25	M	68	Floor of mouth	SCC	4	2	x	Moderate	–	–	Yes	Yes	Rad	6m	Dead
OC26	F	56	Retromolar triangle	SCC	1	0	x	Moderate	–	–	Yes	Yes	Surg+Rad	14m	Dead
OC27	F	51	Lower jaw	SCC	4	1	x	Moderate	–	–	Yes	No	Surgery+Rad	7m	Dead
OC28	M	66	Floor of mouth	SCC	2	0	x	Poor	–	–	Yes	Yes	Surg+Rad	32m+	Alive
OC29	M	57	Tongue	SCC	1	0	x	Well	–	–	No	No	Surg	31m+	Alive
OC30	M	75	Upper jaw	SCC	4	1	x	Moderate	–	–	Yes	Yes	Rad	7m	Dead
OC31	M	62	Lower jaw and Floor of mouth	SCC	4	1	x	Moderate	–	–	Yes	Yes	Surg+Rad	30m+	Alive
OC32	M	67	Tongue	SCC	2	0	x	Moderate	–	–	Yes	No	Surg	30m+	Alive
OC33	M	56	Floor of mouth	SCC	4	0	x	Poor	–	–	Yes	Yes	Rad	6m	Dead
OC34	M	62	Floor of mouth	SCC	2	0	x	Well	–	–	Yes	Yes	Rad	19m	Dead
OC35	M	58	Cheek, upper and lower jaw	SCC	4	2	x	Moderate	–	–	Yes	Yes	No	0	Dead

OC, oral cancer; M, male; F, female; HPV, human papillomavirus strains 16 or 18; SCC, squamous cell carcinoma;

a)age at diagnosis, median age: 60.9 years, age range (48–72 years).

b)T: primary tumour stage.

c)N: regional lymph node stage.

d)M: distant metastasis.

e)Time from diagnosis established until last follow-up or event.

### Selection of the miRNA panel

Twenty-two differentially regulated protein-coding genes were chosen on the basis of a literature review (**Supplementary Table 1**) (28–33). Identification of potential miRNA-binding sites in their 3’UTRs followed by miRNA selection based on complementarity was carried out by miRWalk (http://mirwalk.umm.uni-heidelberg.de). The tool uses data obtained with a machine learning algorithm including experimentally verified miRNA-target interactions. Only miR-21-5p, miR-34b-3p, miR-93-5p, miR-133b, miR-146b-5p, miR-155-5p, and miR-182-5p, miR-193b-5p, miR-429 and miR-518c-3p had binding sites in all 22 protein-coding genes and were kept for further studies.

### Samples processing and RNA extraction

For extraction of total RNA, containing small RNA fraction (>18 nt), from tumor and normal tissue samples, PAXgene Tissue miRNA Kit (Qiagen, Germany) was used. In brief, the PAXgene container stored tissues were carefully wiped in order to remove PAXgene Tissue Stabilizer solution and subsequently mechanically disrupted by a homogenizer in the presence of lysis buffer. The step was followed by centrifugation, precipitation and binding of RNA on the column. Next, DNase treatment and washing were performed according to the manufacturer’s instructions. The exact RNA concentrations were determined spectrophotometrically using NanoDrop2000 (Thermo Scientific) and the integrity was confirmed by Agilent 2100 Bioanalyzer (Agilent Technologies).

### miRNA expression profiling

First, 2 µg of the extracted total RNA contacting miRNAs were polyadenylated and reverse transcribed into cDNA using the miScript Reverse Transcription Kit (Qiagen, Germany) according to the manufacturer’s instructions. In brief, a poly-A tail was added to the mature miRNA template and then reverse transcribed to cDNA by a poly-T primer containing the universal tag. The reverse transcription program included the incubation of the reaction at 37°C for 1 hour, followed by inactivation of the reaction at 95°C for 5 min. Two µl of cDNA (diluted 1:7) was amplified by miRNA-specific forward (2 µl) and universal reverse (2 µl) primers with miScript SYBR^®^ Green PCR Kit (10 µl) (Qiagen, Germany) in total of 20 µl reaction volume. Each sample was run in duplicates for analysis on Rotor-Gene Q real-time PCR detection system (Qiagen, Germany). The amplification protocol included an initial denaturation at 95°C for 15 min, followed by 40 cycles of repetition of the three steps: 94°C for 15 sec, 55°C for 30 sec and 70°C for 30 sec. The expression levels of miRNAs were normalized to RNU6 and SNORD72. The list of miRNAs assays’ ID and catalog numbers are presented in **Supplementary Table 2**. Relative miRNA abundance was calculated using the comparative 2^-ΔΔCt^ method and normalized to the geomean of RNU6 and SNORD72 transcription levels. ΔCt was calculated by subtracting the Ct values of reference genes from the Ct values of the miRNA-of-interest. ΔΔCt was then calculated by subtracting ΔCt of the control from ΔCt of the cancer. The resulting data sets were visualized by MATLAB R2022a.

### HPV detection in tumor specimens

DNA was extracted from tumor tissue samples by using TRI Reagent (Sigma- Aldrich) and analyzed for the presence of 19 medium-high risk HPV strains by the strip hybridization test (Operon, Zaragoza, Spain) according to Mehterov and al., 2021 (26).

### Discrimination power analysis

The diagnostic potential of miR-21-5p, miR-93-5p, miR-133b, miR-146b-5p, miR-155-5p, miR-182-5p in OSCC was evaluated by the receiver operating characteristic (ROC) curve. MATLAB R2022a was used to design ROC curves by comparing the expression levels of the above mentioned miRNA signature in both normal and OSCC tissues from the TCGA cohort. The data generated by the software include parameters such as sensitivity, specificity, area under the curve (AUC), and p-value.

### OSCC TCGA dataset

The data of OSCC patient cohorts of the Broad Institute TCGA Genome Data Analysis Center (http://gdac.broadinstitute.org/ (accessed on 28 January 2016)): Firehose stddata:2016_01_28. Broad Institute of MIT and Harvard (OSCC N = 354 and Normal N = 44) was used to evaluate miR-21-5p, miR-93-5p, miR-133b, miR-146b-5p, miR-155-5p, miR-182-5p in both oral cancer tissues and normal adjacent samples. Wilcoxon test was applied to determine the significance of the miRNA panel and gene modulation between expression values of normal and tumor samples. Significance was defined at the p < 0.05 level.

### miRNA target prediction and pathway enrichment analysis

miRNA-target interaction and pathway enrichment analysis were established by MiRNet (https://www.mirnet.ca/miRNet/home.xhtml (accessed on 1 May 2021)) web tool, based on Tarbase v8. The significance of negative association on patient samples for each validated miRNA panel-target interaction was established by Pearson’s correlation coefficient. All analyses were run on MATLAB R2022a (The MathWorks Inc., Massachusetts, MA, USA, http://www.mathworks.com).

### Survival analyses

Kaplan–Meier analysis was performed to generate data concerning OS and DFS and the differences between curves were assessed by log-rank test. The effects of clinical variables on survival analysis were evaluated by a multivariate Cox proportional hazards regression model. The positive and negative z-score values defined patients with high and low signal intensity. The analyses were conducted entirely with MATLAB R2022a.

## Results

### Identification of OSCC-specific miRNA signature

**Figure 1:**
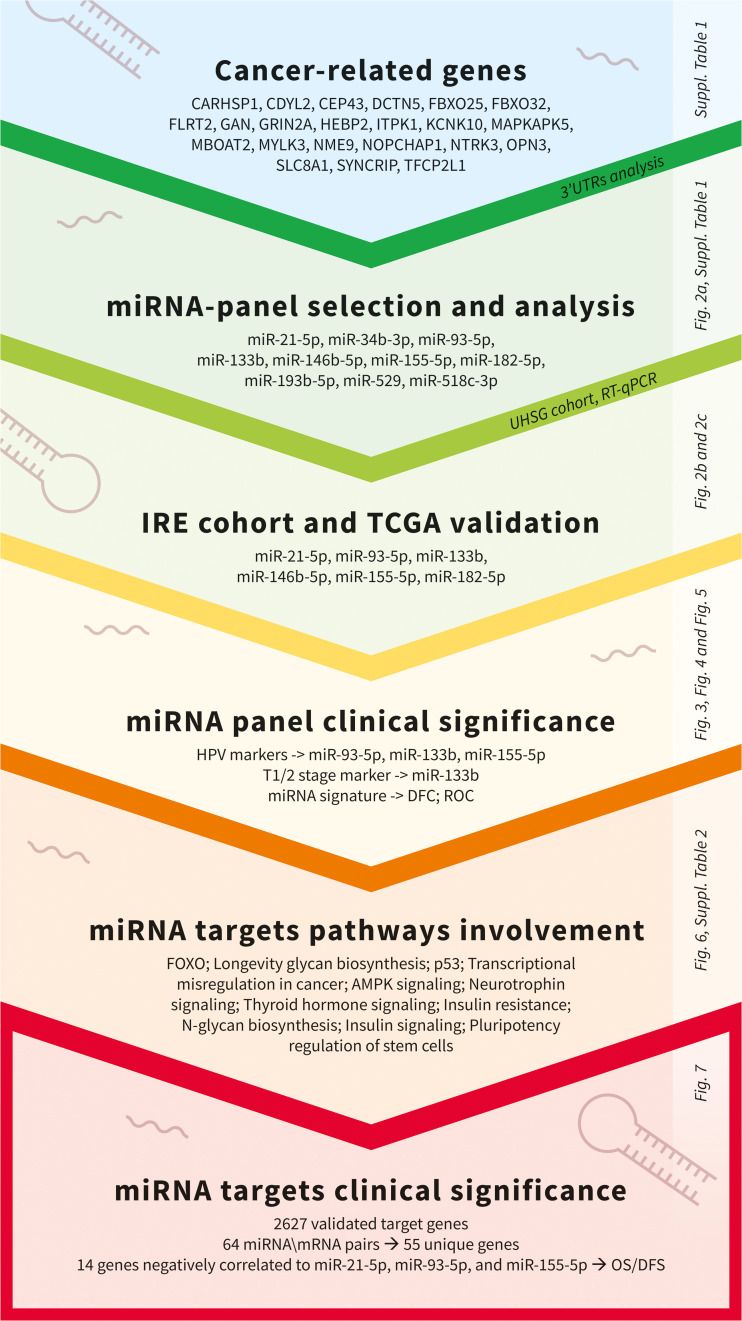
Experimental workflow used for the identification of clinically significant miRNAs and associated targets in OSCC.

**Figure 2:**
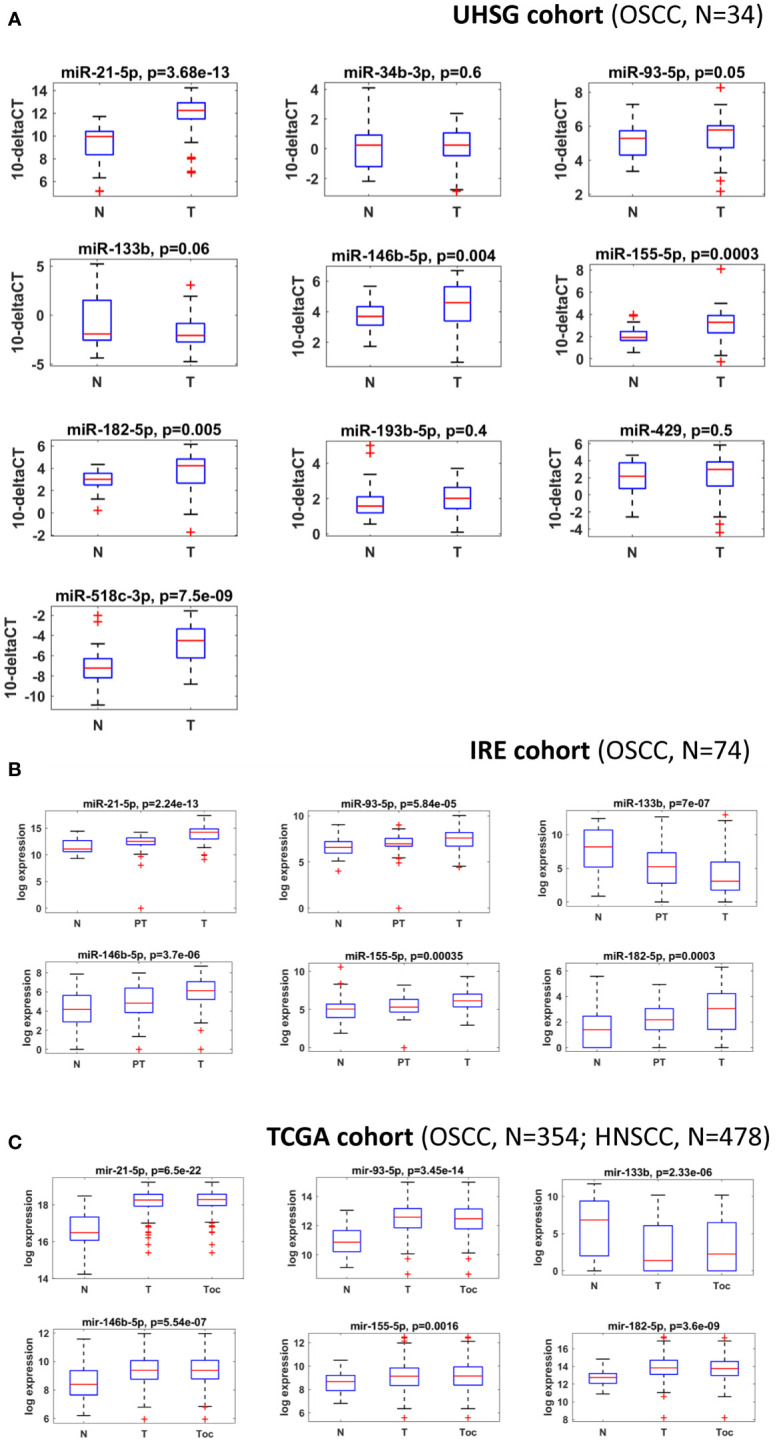
miR-21-5p, miR-93-5p, miR-146b-5p, miR-155-5p, miR-182-5p and miR-133b are differentially expressed in three different OSCC patient cohorts. **(A)** Box plots of the normalized delta Ct expression of a 10 miRNA panel, evaluated in both OSCC and normal tissues from the UHSG cohort. Expression levels were determined by RT-qPCR and presented as fold differences. The reference control genes RNAU6 and SNORD72 were measured with two replicates in each PCR run, and the average Ct value was used for relative expression analysis. Relative miRNA abundance was calculated using the comparative 2^-ΔΔCt^ method and normalized to geomean of the reference gene levels. Data are summarized from two technical replicates for each patient. N=normal, T=Tumoral. **(B)** Box plot showing the expression levels of miR-21-5p, miR-93-5p, miR-146b-5p, miR-155-5p, miR-182-5p, and miR-133b in normal, peritumoral and tumoral OSCC tissues from IRE cohort. N=normal, PT=Periumoral T=Tumoral. **(C)** Box plots showing expression levels of miR-21-5p, miR-93-5p, miR-146b-5p, miR-155-5p, miR-182-5p, and miR-133b in the tumoral and normal OSCC tissues from the TCGA cohort. P ≤ 0.05 was considered statistically significant for all assays. N=normal, T=HNSCC, Toc= OSCC.

miRNAs perform their crucial role as regulators of gene expression mainly by binding to 3’UTRs of their target genes (16, 34). In order to identify OSCC-specific miRNA signatures, we combined both bioinformatical and molecular studies presented as an experimental workflow on **Figure 1**. First, we used a bioinformatical approach through which we analyzed 3’UTRs of 22 oral and other cancers-associated genes (CARHSP1, CDYL2, CEP43, DCTN5, FBXO25, FBXO32, FLRT2, GAN, GRIN2A, HEBP2, ITPK1, KCNK10, MAPKAPK5, MBOAT2, MYLK3, NME9, NOPCHAP1, NTRK3, OPN3, SLC8A1, SYNCRIP, TFCP2L1) (**Figure 1**; **Supplementary Table 1**). As a result, we created a panel of 10 miRNAs (miR-21-5p, miR-34b-3p, miR-93-5p, miR-133b, miR-146b-5p, miR-155-5p, miR-182-5p, miR-193b-5p, miR-429 and miR-518c-3p) that have binding sites in 3’UTRs of all 22 genes analyzed. We proposed that selected miRNAs might show aberrant expression in OSCC versus normal adjacent samples, making them potential tools with diagnostic and prognostic values. As a next step, we profiled the transcription levels of the selected ten miRNAs in OSCC HPV (–) matched tissue samples (n=34) from the UHSG cohort (Bulgaria) using real-time PCR. The obtained Ct values are available in **Supplementary Table 3**. The expression of these miRNAs is presented as boxplots in **Figure 2A**. Using a cut-off value of the 2-fold difference, we identified that the transcriptional levels of miR-21-5p (p < 0.0001), miR-93-5p (p < 0.0197), miR-146b-5p (p < 0.0012), miR-155-5p (p < 0.0001), miR-182-5p (p < 0.0001) were significantly increased, whereas miR-133b (p < 0.05) was decreased in the analyzed tissue samples. In order to confirm our results in different patient cohorts, the expression levels of differentially modulated miRNAs were evaluated in matched tumoral (T), peritumoral (PT), and normal (N) tissues from OSCC patients (n=74) of Regina Elena National Cancer Institute (IRE cohort), Rome, Italy, previously described by Ganci and colleagues (27), and Di Agostino and colleagues (35). Our validation studies showed the same transcriptional behavior for miR-21-5p, miR-93-5p, miR-146b-5p, miR-155-5p, miR-182-5p and miR-133b as primarily identified at UHSG cohort (**Figure 2B**). The final validation in both tumor and normal tissues from TCGA OSCC (n=354) cohort confirmed the results obtained from UHSG and IRE cohorts (**Figure 2C**). Thus, the qPCR results acquired by the analysis of the transcription profiles of miR-21-5p, miR-93-5p, miR-146b-5p, miR-155-5p, miR-182-5p and miR-133b in the UHSG cohort were further validated in two independent patient cohorts (IRE and TCGA). In conclusion, these results suggested that miR-21-5p, miR-93-5p, miR-146b-5p, miR-155-5p, miR-182-5p, and miR-133b are differentially expressed in OSCC tumor tissues in comparison to normal mucose.

### HPV, stage-, gender- and age-dependent miRNAs in OSCC

**Figure 3:**
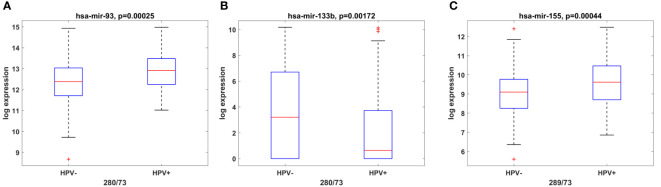
miR-93-5p, miR-133b and miR-155-5p have HPV-dependent expression. Box plots showing the expression levels of miR-93-5p **(A)**, miR-133b **(B)** and miR-155-5p **(C)** in both OSCC HPV+ and HPV- OSCC tissues from the TCGA cohort. P ≤ 0.05 was considered statistically significant for all assays.

Among the classical etiological factors for the development of OSCC such as excessive alcohol consumption and tobacco smoking, HPV infection has gained particular attention not only for oropharyngeal tumors but also for oral cavity cancers. It has been found that HPV positive (HPV+) tumors have distinct miRNA profiles compared to HPV negative (HPV (–)) ones (36, 37). As HPV(+) tumors offer better OS in oral cancer, we decided to investigate whether miRNAs from the panel might have HPV-dependent expression, thus serving as an indirect marker for the presence of the virus. Hence, we performed TCGA analysis of both OSCC HPV(+) and HPV(-) tumors and identified miR-93-5p (**Figure 3A**) and miR-155-5p (**Figure 3C**) to be over-expressed, whereas miR-133b was underexpressed in HPV(+) in comparison to HPV(-) OSCC tissues (**Figure 3B**).

**Figure 4:**
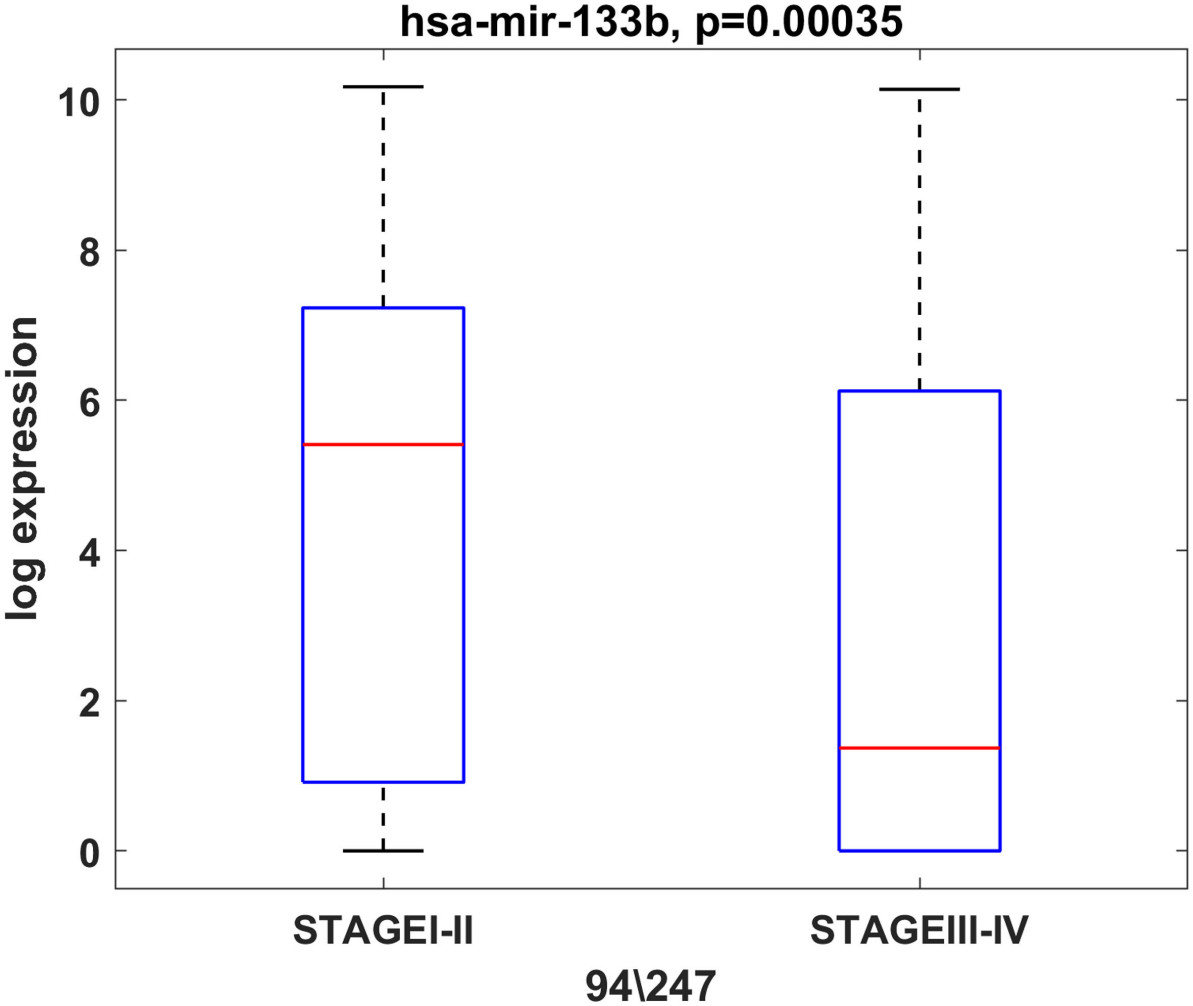
miR-133b is predominantly expressed in initial stage tumors (T1, T2). Box plot showing the expression levels of miR-133b in initial (T1, T2) and advanced (T3, T4) stage tumors from the TCGA cohort. P ≤ 0.05 was considered as a statistically significant value.

In addition, we observed an association between miR-133b expression and the tumor stage. The results showed that miR-133b was highly expressed in stage I and II patients but lowered in stage III and IV patients in TCGA (**Figure 4**). However, the same analysis performed on UHSG and IRE cohorts did not show statistical significance, most probably due to the smaller number of patients included in the study (data not shown). Furthermore, we compared the expression of the initially selected 10 miRNAs between the male (N=30) and female (N=4) UHSG OSCC cohort. Our results showed that miR-34b-3p and miR-93-5p were predominantly expressed in tumor tissues of male OSCC patients (**Supplementary Figure 1**). In order to identify age-dependent miRNA changes, the median age of 60.9 years was estimated (range: 48–72 years) for the UHSG OSCC cohort. Subsequently, we compared the expression of a 10 miRNAs panel in OSCC patients bellow (48-60.9 years) and above the median age (60.9-72 years). As a consequence of this analysis, we showed that miR-182-5p and miR-429 were more expressed in tumors obtained from patients above the median age (**Supplementary Figure 2**).

Altogether, among the validated miRNAs from the OSCC panel, three of them (miR-93-5p, miR-133b and miR-155-5p) could be used to differentiate HPV(+) from HPV(-) tumors, whereas miR-133b could be regarded also as a stage-dependent marker. Moreover, miR-34b-3p and miR-93-5p showed male-dependent OSCC expression pattern. miR-182-5p and miR-429 had higher expression in patients above the median age.

### miRNA signature has prognostic and biomarker properties in OSCC

**Figure 5:**
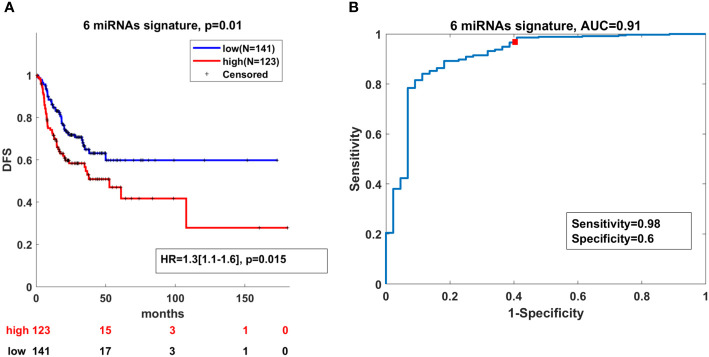
High expression of the miRNA signature correlates with lower DFS and can distinguish OSCC from normal cases. **(A)** Kaplan–Meier analysis representing the correlation between expression levels of the miRNA signature and DFS in OSCC patients from the TCGA cohort. The TCGA cohort was divided in two subgroups according to high and low mean expression levels of each miRNA. High and low subgroups were established by evaluating positive and negative z-scores of the mean expression values of the miRNAs, respectively. For each KM curve, the hazard risk, confidence interval, and relative p-value (p) of the multivariate Cox analysis are also indicated. Cox regression was adjusted for T, N stage and HPV status. **(B)** Diagnostic ability of the miRNA signature for OSCC. ROC curve analysis of the miRNA signature in discriminating between patients and healthy individuals from the TCGA cohort.

Next, we investigated whether these six miRNAs had clinical significance for DFS in OSCC when they were considered as a signature. We assigned a score to each patient based on the expression levels of the six miRNAs from OSCC TCGA. Then, based on the expression levels of the miRNA signature, a Kaplan–Meier analysis was carried out comparing patients with high and low miRNA signature expression values. As presented in **Figure 5A**, the survival analysis showed that high expression of the miRNA signature determined shorter DFS in the TCGA cohort, compared to patients with low expression. To determine whether the selected six miRNAs can distinguish tumor from normal tissues, a ROC curve was constructed to evaluate the discrimination power of this signature in OSCC diagnosis. The designed ROC curve showed that the miRNA signature can efficiently distinguish OSCC cases from controls with areas under the curve (AUC) of 0.91 with sensitivity and specificity of 0.98 and 0.6, respectively (**Figure 5B**).

Taken together, these findings demonstrate that the expression levels of the miRNA signature had prognostic power related to DFS and held biomarker properties in OSCC.

### miR-21-5p, miR-93-5p, miR-146b-5p, miR-155-5p, miR-182-5p and miR-133b modulated genes in OSCC

**Figure 6:**
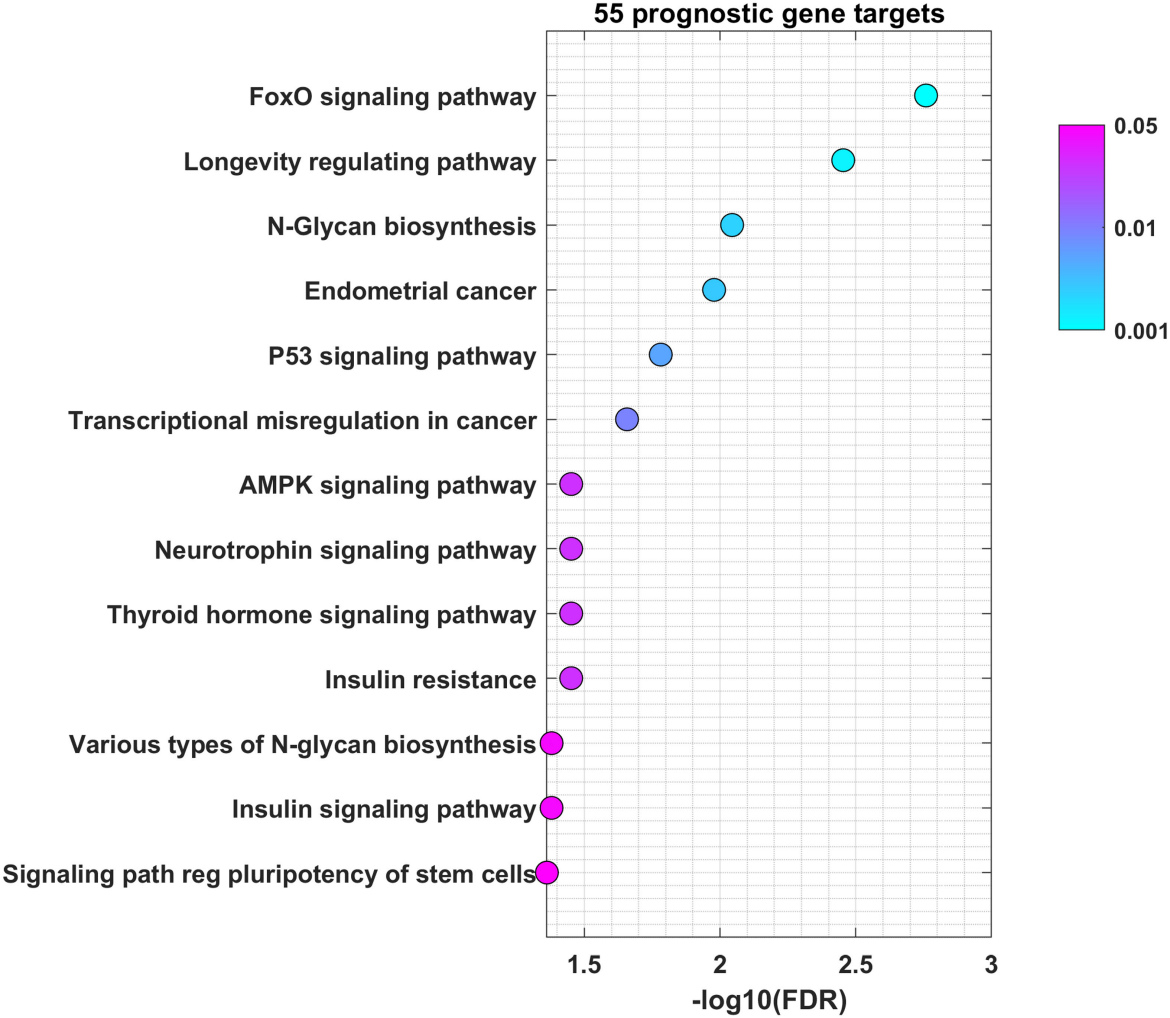
miR-21-5p, miR-93-5p, miR-146b-5p, miR-155-5p, miR-182-5p and miR-133b control genes involved in FOXO, longevity, glycan biosynthesis and p53 pathways in OSCC. Bubble plot containing specific ontological groups of miR-21-5p, miR-93-5p, miR-146b-5p, miR-155-5p, miR-182-5p and miR-133b validated targets. The graphs represents only the GO groups above the established cut-off criteria (p with correction <0.05, minimal number of genes per group >10). Each bubble displays the number of differentially expressed genes assigned to the particular GO terms. The transparency of the bubbles shows the p-values (the darker the violet color, the closer to the border of p = 0.05).

To understand through which genes miRNAs mediate their role in OSCC, we used miRNet for selection of validated protein-coding genes regulated by miR-21-5p, miR-93-5p, miR-146b-5p, miR-155-5p, miR-182-5p and miR-133b. Our analysis led to the identification of 2627 validated target genes (**Supplementary Table 4**). This was followed by the identification of 64 miRNA\mRNA pairs for a total of 55 unique genes. Each gene showed negative correlation to the selected miRNAs. The biological processes regulated by the selected targets are shown in **Figure 6**. The results of this functional enrichment analysis revealed significant enrichment in 13 GO processes (p < 0.05). The pathways which were mostly involved included FOXO, longevity, glycan biosynthesis and p53. In conclusion, the common genes regulated by miR-21-5p, miR-93-5p, miR-146b-5p, miR-155-5p, miR-182-5p and miR-133b were implicated in different cancer-related pathways such as FOXO, longevity, glycan biosynthesis and p53.

### miR-21-5p, miR-93-5p, and miR-155-5p targets had clinical significance in OSCC

**Figure 7:**
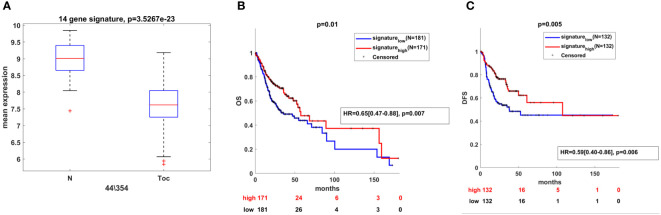
Clinical utility of miR-21-5p, miR-93-5p, and miR-155-5p targets in OSCC. **(A)** Box-plot representing the expression of 14 genes negatively correlated with miR-21-5p, miR-93-5p, and miR-155-5p and differentially expressed between oral tumor tissues and their normal counterparts (P<0.05) in the TCGA OSCC cohort. Kaplan–Meier analysis representing the correlation between expression levels of miR-21-5p, miR-93-5p, and miR-155-5p targets considered as a group and OS **(B)** and DFS **(C)** in OSCC patients from the TCGA cohort. The TCGA cohort was divided in high and low groups, based on the positive and negative z-scores of the mean signal of the signature, respectively. The Hazard Ratio was assessed by multivariate cox regression analysis, adjusted for T,N stage and mutP53. For each KM curve, the hazard risk, confidence interval, and relative p-value (p) of the multivariate Cox analysis are also indicated.

To explore the clinical significance of the deregulated miRNAs from the panel in OSCC, we combined the information of their expression levels with the expression profiles of their targets obtained by TCGA. For that purpose, the list of 55 unique genes used for pathway identification analysis was further shrank to 14 genes by the application of Fold Change<-1.5 and Pearson’s correlation coefficient higher than 0.3 in absolute value (**Table 2**). Each of the 14 genes negatively correlated to the miR-21-5p, miR-93-5p, and miR-155-5p signature as shown on **Figure 7A**. In addition, the Kaplan–Meier analysis revealed the clinical utility of the 14 genes related to both OS (**Figure 7B**) and DFS (**Figure 7C**) when considered as a group. As it can be seen in **Figures 7B, C**, the survival analysis demonstrated that the high expression of miR-21-5p, miR-93-5p, and miR-155-5p targets (gene signature high) was predictive of shorter OS (**Figure 7B**) and DFS (**Figure 7C**) in the TCGA cohort, compared to patients with negative scores (gene signature low). These results underlined the prognostic utility of miR-21-5p, miR-93-5p, and miR-155-5p targets when used as a signature in OSCC patients.

**Table 2:** miR-21-5p, miR-93-5p, and miR-155-5p validated targets, expression levels and OS/DFS significance.

miR	validated target	Pearson R	p	logFold T\N	Wilcoxon p	prognostic
hsa-mir-21-5p	ABCB1	-0,30394	5,96E-10	-1,35672	7E-09	OS\DSF
hsa-mir-93-5p	CCNG2	-0,31063	2,37E-10	-1,014	4,48E-11	DFS
hsa-mir-93-5p	CLOCK	-0,30966	2,72E-10	-0,75696	9,92E-08	DFS
hsa-mir-93-5p	FCHO2	-0,35486	2,96E-13	-1,0879	5,32E-14	OS
hsa-mir-93-5p	GRID2IP	-0,40326	5,38E-17	-2,16724	2,78E-17	OS
hsa-mir-93-5p	GTF2IRD2	-0,33952	3,4E-12	-1,05955	4,59E-14	OS
hsa-mir-93-5p	KAT2B	-0,49641	3,76E-26	-1,93361	1,06E-20	OS\DFS
hsa-mir-93-5p	KLB	-0,44141	2,08E-20	-2,52994	4,03E-17	OS
hsa-mir-155-5p	PELI1	-0,30125	8,56E-10	-0,68675	7,36E-07	DFS
hsa-mir-21-5p	PHACTR2	-0,35428	3,26E-13	-0,82007	7E-09	DFS
hsa-mir-93-5p	RAB11FIP1	-0,38855	8,59E-16	-1,63602	5,85E-15	DFS
hsa-mir-93-5p	RMND5B	-0,43373	1,1E-19	-1,29803	1,44E-19	OS
hsa-mir-93-5p	SASH1	-0,49166	1,3E-25	-2,24475	1,17E-20	DFS
hsa-mir-93-5p	SESN1	-0,36487	5,61E-14	-0,90881	3,11E-11	OS
hsa-mir-21-5p	TLR4	-0,34258	2,11E-12	-0,65603	0,011507	DFS

## Discussion

As in other malignancies, oral carcinogenesis is a multistep process caused by gradual accumulation of both genetic and epigenetic alterations (38, 39). As a result, the gain of function in specific oncogenes and the loss of function in some tumor-suppressor genes cause cell proliferation in an uncontrolled way (40). In the last decade, an increasing amount of evidence has suggested that some miRNAs might have either an oncogenic or a tumor-suppressor role. This evidence, together with their gene regulatory functions, makes them suitable targets for oral cancer investigations. Moreover, the expression profile of many miRNAs is pretty stable in tissue and blood samples of cancer patients. Therefore, they hold promise as essential biomarkers in oral cancer. Driven by the importance of early cancer diagnosis and the need to distinguish between malignant and benign tumor types, the scientific efforts are directed towards identification and validation of a panel of miRNAs to diagnose the disease and monitor its progression. Thus, the understanding of abnormal miRNA expression would help in the identification of novel markers and drug targets in OSCC.

In the present study we found tumor-specific modulation in the expression of miR-21-5p, miR-93-5p, miR-146b-5p, miR-155-5p, miR-182-5p and miR-133b in three different patient cohorts: UHSG, IRE and TCGA. Among them, miR-21-5p is a very well-known oncomiR, reported in oral (27, 41–44) and in many other malignancies, including colorectal cancer (45), esophageal cancer (46), hepatocellular cancer (47, 48), gastric cancer (49), lung cancer (50), breast cancer (51), cervical cancer (52) and bladder cancer (53). The restriction in the expression of miR-21 *in vitro* and *in vivo* leads to growth inhibition, reduced proliferation and activation of apoptosis (41, 44). In addition, miR-21-5p, together with miR-21-3p, miR-96-5p, miR-429 are differentially expressed in the peritumoral region and can actually predict local recurrence independently of prognostic clinical variables, either when considered individually or as a group (27). miR-93-5p, the second miRNA from our panel, is weekly investigated in OSCC. D’Souza and Kumar reported that miR-93, together with 13 other miRNAs, is upregulated in oral cancer (54). The same transcriptional behavior was also observed in few other studies, even though miR-93 was not the main focus (55–57). miR-146b has oncogenic properties in OSCC and is able to bind HBP1 directly, leading to increased proliferation, migration, and invasion of OSCC cells (58). The suppressive effect of miR-146b inhibition was further abolished by co-inhibition of HBP1. In addition, miR-146b-5p, together with miR-31, miR-142-5p, miR-33a, miR-1259, miR-146b-5p, miR-886-3p, miR-886-5p, miR-519d, and miR-301a have been shown to be upregulated in malignantly transformed oral leukoplakia but not in oral leukoplakia tissue samples (59). Several authors reported that miR-155-5p promotes the progression and surveillance of oral cancer by modulation of different targets such as the chromatin remodeling gene ARID2 and TP53INP1 that confirms resistance to 5-Fluorouracil (60). Moreover, the high tissue expression of miR-155-5p is associated with poor outcome and cervical lymph nodes metastasis (61, 62). Similarly to miR-21-5p and miR-155-5p, miR-182-5p also acts as an oncogene in OSCC. This miRNA has high expression in oral cancer cell lines and tissue samples (63). The downstream target of miR-182-5p, CAMK2N1, acts as a tumor suppressor, through which this miRNA controls the growth, proliferation and colony formation abilities in OSCC (63). Circ_0000140 sponges miR-182-5p which in turn recovers the expression of CDC73, followed by impaired proliferation, metastasis and glycolysis of OSCC (64). Also, miR-182-5p increases the migratory and invasion properties of OSCC through binding with MTSS1 (65). The last miRNA investigated, miR-133b has been reported by Yang and collaborators (66) to act as a tumor suppressor. Its lower transcription levels corelate with worse prognosis in OSCC patients. The artificial substitution with miR-133b-mimics in SCC9 cells reduced their growth and metastasis. The same study showed SOX4 as a direct target of miR-133b (66). Another investigation, performed on head and neck tumor tissues and cancer cell lines, proved that the expression of miR-133b negatively correlated with Nup214 (67). The ectopic expression of miR-133b decreased the levels of Nup214, increased mitotic indices and delayed degradation of mitotic marker proteins such as cyclinB1 and cyclinA. The observed mitotic delay enhanced chromosomal abnormalities and apoptosis (67).

Even though the presence of HPV suggests better survival outcome, there is no significant difference in the five-year survival rates between HPV-positive and HPV-negative HNSCC patients (68). Also, the treatment and clinical approach differs between the two patient groups. Undoubtedly, new biomarkers are urgently needed for the discrimination between HPV(+) and HPV(-) patients which will improve survival rates and minimize treatment toxicity. We have identified miR-93-5p (p < 0.0003), miR-133b (p < 0.0017) and miR-155-5p (p < 0.0004) that can discriminate HPV(-) from HPV(+) OSCC patients. The transition of early (T1+2) to late (T3+4) tumor stages is accompanied by drastic changes at genetic and epigenetic levels. Our investigation demonstrated that as tumor stage increased, miR-133b expression decreased, suggesting that this miRNA could be used as a stage-dependent marker. In addition, some of the miRNAs investigated (miR-34b-3p and miR-93-5p) showed sex-dependent OSCC expression, being more abundant in OSCC tumors of male patients. Furthermore, miR-182-5p and miR-429 were predominantly expressed in OSCC patients above the estimated median age (60.9-72 years). Our findings underscored the clinical significance of the used miRNA panel as 1) it could efficiently distinguish OSCC cases from controls with AUC of 0.91 (sensitivity=0.98; specificity=0.6) and 2) high expression of the miRNA signature determines shorter DFS in the TCGA cohort as demonstrated by Kaplan–Meier curves.

Cancer cells are characterized by aberrant regulation of many signaling pathways that precede tumorigenesis and impose drug resistance (69). Our study found that most of miR-21-5p, miR-93-5p, miR-146b-5p, miR-155-5p, miR-182-5p and miR-133b targets were prevalent in the FOXO signaling pathway. The FOXO family transcription factors contains four members FOXO1, FOXO3a, FOXO4 and FOXO6 in mammals (70, 71). FOXO factors regulate a variety of downstream genes with tumor suppressor properties, such as FasL, 15, p19, NOXA, FasL, TRAIL and Bim controlling apoptosis and p27 and cyclin D that govern the cell cycle (72–77). Moreover, FOXO1 induces authophagy–mediated cell death as well as inhibits Runx2-mediated cell migration and invasion that are not related to its transcription activity (78, 79). Also, FoxO transcription factors influences almost every aspect of T-cell biology. They respond to a wide spectrum of extrinsic signals to alter the T-cell dependent immune response (80). T and B cells express FoxO1 and FoxO3, respectively. It has been shown that FoxOs are crucial for T cell homeostasis (81). FoxO1 is essential for the regulation of several genes involved in T-cell survival. They also play a critical role in the differentiation of memory CD8+ T cells. Many studies emphasize the essential role of FoxO in regulating specialized lymphocyte functions. FoxO1 also controls the proliferation, differentiation and survival of B cells (80). FoxO1 is generally found in its inactive form in dendritic cells, which warrants their survival and proliferation (82, 83). Moreover, FoxO1 stimulates both pro- and anti-inflammatory pathways in macrophages (84, 85) Those data identify FoxO as a major regulator of immune cells which imposes the implication that our findings could reflect the immune processes developing within the tumor tissue. All this data suggests that FOXO1 as well as the other FOXO members could be used as therapeutic targets in anticancer research (86–90). N-glycan biosynthesis is another pathway in which the targets of the analyzed signature participate. Cancer progression and metastasis are accompanied by massive glycosylation of many proteins such as adhesion proteins or proteases (91). As local invasiveness and high risk of lymph node metastasis represent two of the main hallmarks of OSCC, it is not surprising that miR-21-5p, miR-93-5p, miR-146b-5p, miR-155-5p, miR-182-5p and miR-133b targets actively participate in N-glycan biosynthesis. Finally, we underlined the clinical significance of 14 targets of miR-21-5p, miR-93-5p, and miR-155-5p. Their high expression as a signature determined shorter OS and DFS in the TCGA cohort, compared to patients with low gene signature expression.

## Conclusion

Our research was driven by the fact that reliable biomarkers for oral cancer diagnosis and prognosis are still missing. For this reason, the scientific efforts are directed towards the identification of stable and reproducible biomarkers, as miRNA expression profiles. Although the choice of the selected miRNAs is based on *in silico* analysis, in the present work we identified a new panel of six miRNAs (miR-21-5p, miR-93-5p, miR-146b-5p, miR-155-5p, miR-182-5p and miR-133b) differently expressed in OSCC tissue samples. Their modulation was confirmed in three different patient cohorts. Through the miRNet analysis, we have attributed the possible role of the identified miRNAs in OSCC tumorigenesis by using specific cut off criteria. Based on them, we focused on specific targets but maybe lost some others that could have an important role in the neoplastic process. Another limitation of our study is the fact that we did not provide any direct evidences for the proposed miRNA-mRNA interactions. Overall, our results demonstrate that miR-133b could differentiate T1/T2 tumor stages from T3/T4, whereas miR-93-5p, miR-133b and miR-155-5p are markers for HPV-induced tumors. The results of the performed ROC curve analysis and survival studies confirmed the robustness of the proposed miRNA panel. The clinical relevance of the identified signature should be further validated in a multicentric observational study. This will allow clinicians to have a valuable tool for OSCC patient management. We believe that it could have a clinical value and could possibly help the use of these miRNAs as reliable biomarkers in OSCC.

## Data availability statement

The microarray data sets used in the current study concerning IRE are submitted to GEO database with an accession ID: GSE216630. TCGA data (https://portal.gdc.cancer.gov/) are publicly available. 

## Ethics statement

The studies involving human participants were reviewed and approved by Institutional Ethics Committee of Medical University-Plovdiv (Protocol №1/25.02.2016). The patients/participants provided their written informed consent to participate in this study.

## Author contributions

The authors confirm contribution to the paper as follows: study conception and design: NM, AS. Data collection: NM, AS, CP. Analysis and interpretation of results: NM, AS, GB, IB-N, BV, VS. Sample collection and analysis: NM, BN, GH, DP, BV. Draft manuscript preparation: NM; review and editing: GB, IB-N, BV, VS. All authors contributed to the article and approved the submitted version.

## Funding

This research was funded by Bulgarian Ministry of Science, grant numbers KP-06-M23/1, Project NUCBAS-BBMRI.BG, Contract D01-285/17.12.2019 (Ministry of Education and Science) within the frame of the Bulgarian National Roadmap for Research Infrastructure and partially funded by Medical University – Plovdiv (projectПМД-02/2022). GB acknowledges the support of the Fondazione AIRC under IG 2017, grant ID 20613.

## Acknowledgments

The authors thank Kristin Ozanian for the art work.

## Conflict of interest

The authors declare that the research was conducted in the absence of any commercial or financial relationships that could be construed as a potential conflict of interest.

## Publisher’s note

All claims expressed in this article are solely those of the authors and do not necessarily represent those of their affiliated organizations, or those of the publisher, the editors and the reviewers. Any product that may be evaluated in this article, or claim that may be made by its manufacturer, is not guaranteed or endorsed by the publisher.

## Supplementary material

Supplementary Table 1 List of 22 protein-coding genes used for the selection of 10 miRNA panel.

Supplementary Table 2 The list of 10 miRNAs panel assays ID and catalog number.

Supplementary Table 3 RT-qPCR expression values of miR-21-5p, miR-34b-3p, miR-93-5p, miR-133b, miR-146b-5p, miR-155-5p, miR-182-5p, miR-193b-5p, miR-429 and miR-518c-3p of 34 patients from UHSG cohort.

Supplementary Table 4 miR-21-5p, miR-93-5p, miR-146b-5p, miR-155-5p, miR-182-5p and 453 miR-133b validated target genes used for functional enrichment analysis (**Figure 6**) and OS/DFS 454 (**Figure 7**).

Supplementary Figure 1 Box plots of the normalized delta Ct expression of miR-21-5p, miR-34b-3p, miR-93-5p, miR-133b, miR-146b-5p, miR-155-5p, and miR-182-5p, miR-193b-5p, miR-429 and miR-518c-3p in both male and female OSCC patients from UHSG cohort.

Supplementary Figure 2 Box plots of the normalized delta Ct expression of miR-21-5p, miR-34b-3p, miR-93-5p, miR-133b, miR-146b-5p, miR-155-5p, and miR-182-5p, miR-193b-5p, miR-429 and miR-518c-3p in OSCC patients bellow (48-60.9 years) and above the median age (60.9-72 years) from UHSG cohort.
