# Supplementary figures and images for: A novel panel of clinically relevant miRNAs signature accurately differentiates oral cancer from normal mucosa
Source: Front Oncol. 2022 Dec 1;12:1072579. doi: 10.3389/fonc.2022.1072579 (PMC9753689; doi:10.3389/fonc.2022.1072579)

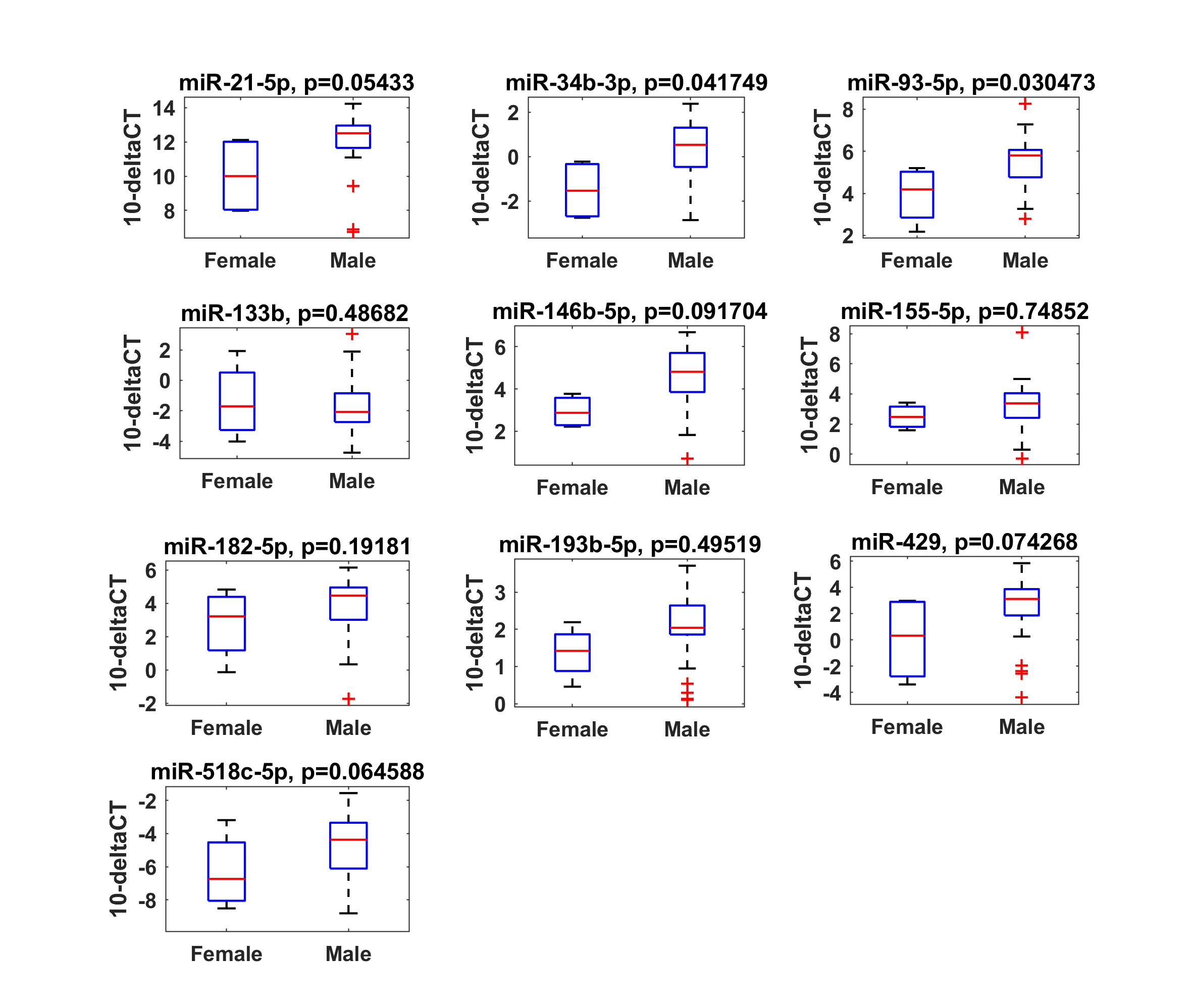

Supplement: Supplementary Figure 1 — Box plots of the normalized delta Ct expression of miR-21-5p, miR-34b-3p, miR-93-5p, miR-133b, miR-146b-5p, miR-155-5p, and miR-182-5p, miR-193b-5p, miR-429 and miR-518c-3p in both male and female OSCC patients from UHSG cohort. [file Image_1.png]

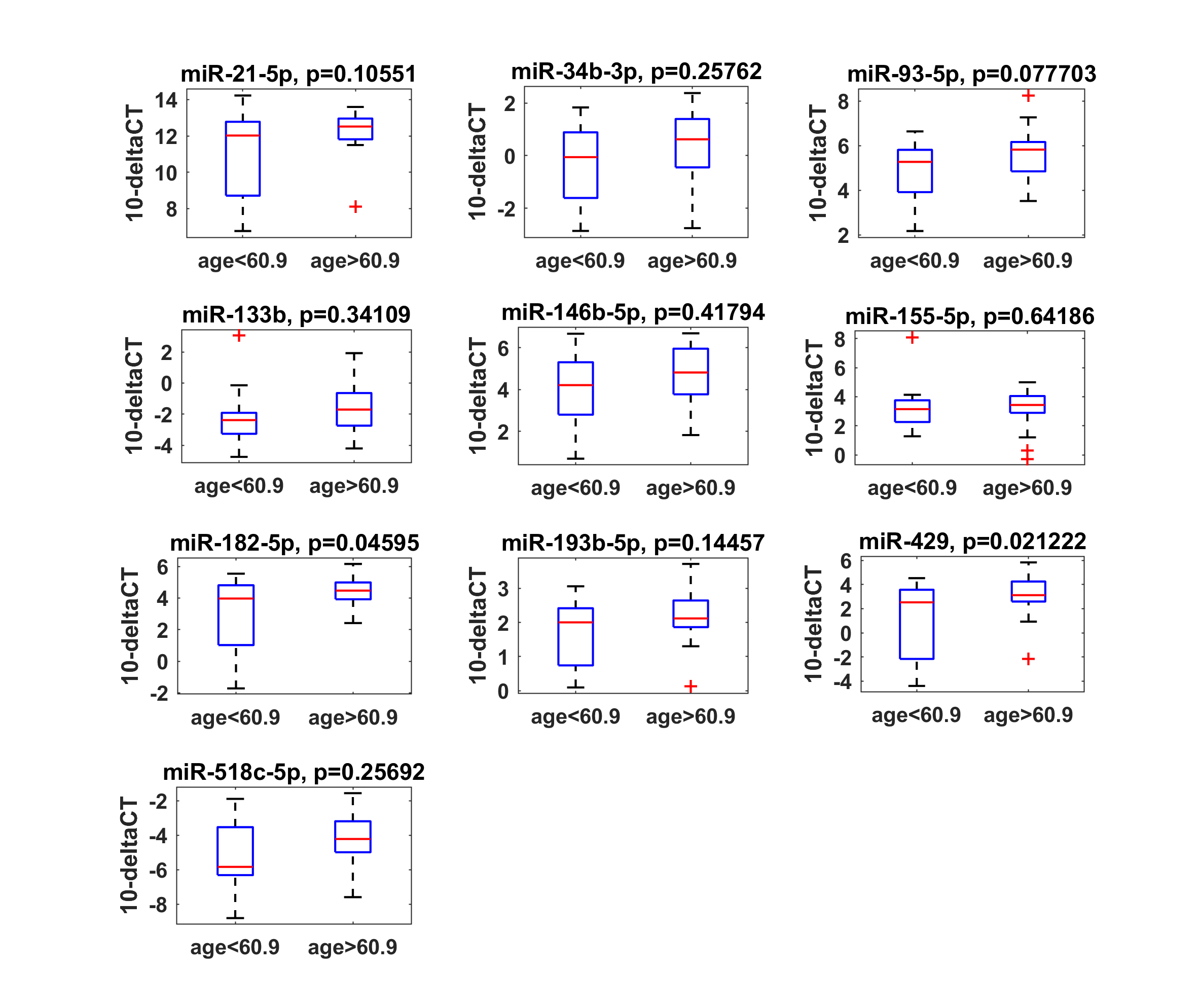

Supplement: Supplementary Figure 2 — Box plots of the normalized delta Ct expression of miR-21-5p, miR-34b-3p, miR-93-5p, miR-133b, miR-146b-5p, miR-155-5p, and miR-182-5p, miR-193b-5p, miR-429 and miR-518c-3p in OSCC patients bellow (48-60.9 years) and above the median age (60.9-72 years) from UHSG cohort. [file Image_2.png]
